# Prediction of Short-Term Mortality of Cardiac Care Unit Patients Using Image-Transformed ECG Waveforms

**DOI:** 10.1109/JTEHM.2023.3250352

**Published:** 2023-02-28

**Authors:** Terumasa Kondo, Atsushi Teramoto, Eiichi Watanabe, Yoshihiro Sobue, Hideo Izawa, Kuniaki Saito, Hiroshi Fujita

**Affiliations:** Graduate School of Health SciencesFujita Health University 12695 Aichi 470-1192 Japan; Division of CardiologyDepartment of Internal MedicineFujita Health University Bantane Hospital Aichi 454-8509 Japan; Department of Cardiology, School of MedicineFujita Health University 12695 Aichi 470-1192 Japan; Faculty of EngineeringGifu University 12785 Gifu 501-1193 Japan

**Keywords:** Deep learning, electrocardiogram, GradCAM, mortality prediction

## Abstract

Objective: The early detection of cardiac disease is important because the disease can lead to sudden death and poor prognosis. Electrocardiograms (ECG) are used to screen for cardiac diseases and are useful for the early detection and determination of treatment strategies. However, the ECG waveforms of cardiac care unit (CCU) patients with severe cardiac disease are often complicated by comorbidities and patient conditions, making it difficult to predict the severity of further cardiac disease. Therefore, this study predicts the short-term prognosis of CCU patients to detect further deterioration in CCU patients at an early stage. Methods: The ECG data (II, V3, V5, aVR induction) of CCU patients were converted to image data. The transformed ECG images were used to predict short-term prognosis with a two-dimensional convolutional neural network (CNN). Results: The prediction accuracy was 77.3%. Visualization by GradCAM showed that the CNN tended to focus on the shape and regularity of waveforms, such as heart failure and myocardial infarction. Conclusion: These results suggest that the proposed method may be useful for short-term prognosis prediction using the ECG waveforms of CCU patients. Clinical impact: The proposed method could be used to determine the treatment strategy and choose the intensity of treatment after admission to the CCU.

## I. Introduction

Heart disease is the most common cause of death in the world [1].Severe cardiac disease is associated with sudden death and poor prognosis; therefore, intensive observation and treatment are performed in the cardiac care unit (CCU) [2]. CCU is a specialized ward where coronary diseases such as myocardial infarction and angina pectoris are intensively managed, circulatory dynamics are checked, and emergency examinations are performed 24 hours a day. Because patients admitted to the CCU are acutely ill, early detection of further illnesses and complications is important. Twelve-lead electrocardiography is a useful test for predicting complications and further illness severity in patients with cardiac disease [3], [4], [5]. However, because the electrocardiogram (ECG) waveform on admission to the CCU is affected by the patient’s condition and comorbidities, rapid and accurate diagnosis depends on the cardiologist’s experience and skill [6]. There is concern that inexperienced doctors, such as residents responding to outpatient emergencies, may not be able to make a prompt diagnosis due to the difficulty of deciphering the ECG. Therefore, objective analysis of the ECG waveforms in CCU patients using deep learning and risk assessment may lead to diagnostic support for doctors and avoid serious illnesses and complications in CCU patients.

In recent years, convolutional neural networks (CNN), a type of deep learning, have been applied in various fields including medical image analysis [7], [8], [9], [10], [11], [12], [13]. Many studies using CNN have been conducted ECG analysis [14], [15], [16], [17], [18], [19], [20], [21]. Liu et al. inputted multi-lead ECGs into a CNN to detect myocardial infarction [14]. The results showed that the classification was possible with high accuracy. Sensitivity, specificity, and accuracy were 95.4%, 97.3%, and 96.0%, respectively. Goodfellow et al. used deep convolutional neural networks (DNNs) to classify the normal sinus rhythm, atrial fibrillation, and other rhythms [16]. The results showed that the classification was possible with high accuracy. Precision, recall, and accuracy were 84%, 85%, and 88%, respectively. Studies have also been conducted to predict patient prognosis by inputting ECG waveforms into CNN [4], [22], [23], [24], [25], [26]. Predicting patient prognosis may be useful in determining the intensity of treatment after hospitalization, the need for invasive treatment, and other future treatment strategies. Raghunath et al. attempted to predict survival after one year by inputting 12-lead electrogram waveforms into a DNN [26], the area under the curve (AUC) was 0.88, indicating that it can predict patient prognosis with high accuracy. However, this study has two limitations. The first is that it is a relatively long-term (1 year) prognostic study and not a short-term prognostic study of CCU admissions, which are more difficult. The second is that numerical data are input to the DNN. We believe that a more detailed analysis could be performed by inputting image data, which allows the shape of the ECG waveform to be read more visually.

In recent years, it has also been known that high performance can be obtained when models that learn from natural images are diverted to other applications such as medical image processing [27], [28], [29]. Furthermore, there have been many reports on the conversion of biological signals such as ECG and EEG into images and advanced waveform analysis by CNN. Cho and Jang [30], [31] detected seizures in EEG. They compared three different input methods: raw time series data, two-dimensional images with short-time Fourier transform, and two-dimensional images of raw EEG waveforms. The best results were obtained when raw two-dimensional images were input to the CNN, with an AUC of 0.993 [30]. Ullah et al. converted ECGs to spectrogram images, which are two-dimensional images, and input them into a CNN for automatic classification of arrhythmias. They achieved sensitivity and specificity of 97.9% and 99.6%, respectively, which was superior to other automatic classification methods using one-dimensional waveform data [31]. A more detailed analysis of ECG waveforms can be performed using a pre-trained CNN model and performing transfer learning. Therefore, in this study, we aimed to predict the prognosis of patients with CCU by focusing on image data, which enables a more visual analysis of the shape of the ECG waveforms. Specifically, the main purpose of this study was to develop a method for short-term prognosis prediction using two-dimensional ECG images and the CNN of CCU patients, and to analyze the prediction basis of CNN.

## II. Methods

### A. Overview of the Proposed Method

**FIGURE 1.**
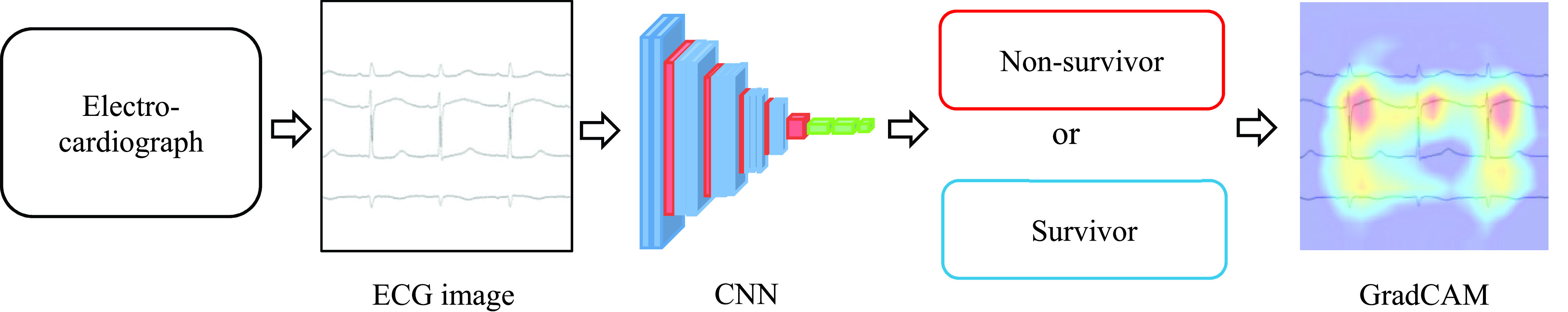
Overview of the proposed method.

An overview of the proposed method is presented in Figure 1. The ECG waveforms were converted into images. The transformed image data were provided to the pretrained CNN, and CNN transfer learning was performed. The prediction accuracy was evaluated using cases that were not used in the training. A color map showing the basis for judgment of the prediction results was obtained using GradCAM [32].

### B. Electro-Cardiogram

**FIGURE 2.**
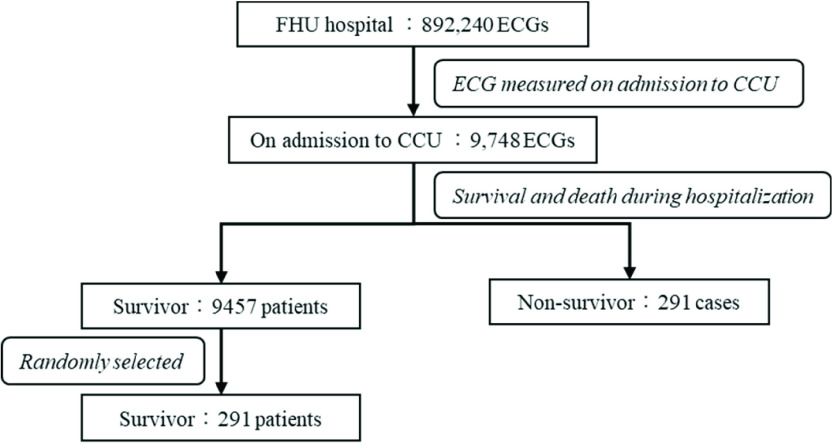
Summary of the data used in the study.

Figure 2 shows a flowchart of the inclusion in this study. 892,240 cases of 10-s resting 12-lead electrocardiograms were measured at Fujita Health University Hospital. Among 9,748 patients admitted to the CCU, there was a mixture of resting and stress ECG waveforms, and waveforms with measurement durations of 10 and 20 seconds. Five thousand potentials were extracted from the ECG waveforms at rest and during the 10 s measurement period. Of these patients, 291 died of cardiac causes during hospitalization at the CCU. In this study, there is a bias in the number of data, with 9457 cases in the survivor group and 291 cases in the non-survivor group. Therefore, 291 cases are randomly sampled from the survivor group in order to prevent the generalization performance of the CNN from deteriorating due to overfitting. We compared the distribution of age, sex, and BMI between the surviving eligible patients and the population of 9,457 CCU patients (surviving), and found that the population, before selection, consisted of 72 ± 13 years of age (mean ± standard deviation), 3489/5968 for sex (male/female), and 22 ± 4 for BMI (mean ± standard deviation). The results of Student’s t-test showed p-values of 0.68, 0.62, and 0.47, respectively, and no statistically significant differences were confirmed. Table 1 lists the baseline clinical characteristics of the cohort and study selection. This study was approved by an institutional review board of Fujita Health University and informed consents were obtained from patients subject to the condition of data anonymization (No. HM19-345).

**TABLE 1:** Baseline Clinical Characteristics

	Nonsurvivor (n = 291)	Survivor (n = 291)	P-value
Age, years (SD)	74±14	72±12	0.13
Sex, male/female	181/110	179/112	0.99
Height, cm (SD)	160±9	159±10	0.222
Weight, Kg (SD)	56±17	58±12	0.325
BMI, $\text{m}^{2}$ (SD)	21±6	21±6	0.446
SBP, mmHg (IQR)	110 (85–136)	136 (116–157)	< 0.001
DBP, mmHg (IQR)	65 (49–86)	79 (66–94)	< 0.001
HR, beat per min (IQR)	89 (72–108)	81 (67–103)	0.098
STEMI	109	38	< 0.001
NSTEMI	25	59	< 0.005
12-lead ECG at admission
RR interval, ms (IQR)	0.67 (0.55–0.83)	0.74 (0.58–0.89)	0.191
PR interval, ms (IQR)	0.16 (0.14–0.19)	0.16 (0.15–0.19)	0.710
QRS interval, ms (IQR)	0.12 (0.10–0.15)	0.10 (0.09–0.12)	< 0.005
QT interval, ms (IQR)	0.39 (0.35–0.44)	0.39 (0.35–0.42)	0.029
QRS axis at admission, degree (SD)	18.8±74.3	27.1±49.6	0.141
SV1, mV (IQR)	0.5 (0.3–0.9)	0.8 (0.5–1.2)	< 0.001
RV5, mV (IQR)	0.9 (0.5–1.4)	1.5 (0.9–2.0)	< 0.001
SV1+RV5, mV (IQR)	1.4 (0.9–2.1)	2.3 (1.6–3.1)	0.942
ST-T change	117	79	< 0.005

BMI, Body mass index; SBP, Systolic Blood Pressure; DBP, Diastolic Blood Pressure; HR, Heart rate; STEMI, ST elevation myocardial infarction; NSTEMI, non-ST elevation myocardial infarction Data are presented as the mean ± SD or number (%) or medians ( $25^{th}$ and $75^{th}$ percentile).

### C. ECG Data Preparation

In this study, the ECG waveform data were converted into images for two-dimensional analysis. The data files extracted from the electrocardiographs were converted into MFER files, a common standard for ECG [33]. Ten seconds of ECG potential were extracted from the MFER file. The extracted cardiac potentials were converted to a grayscale 8-bit PNG format.

In this study, 4-lead ECGs were selected from 12-lead ECGs to the 2-dimensional CNN. From the inductions demonstrated by Raghunath et al. [26] and Park et al. [34] to be highly accurate in predicting survival by DNN, we selected and used the II induction among the I-III inductions, V3 induction among V1-V3, V5 induction among V4-V6, and aVR induction among the aVF, aVR, and aVL induction. The aVR induction was monitored and displayed by performing four arithmetic operations on the electrocardiograph. The formula for calculating the waveform aVR(t) of the aVR induction is shown in (1). I(t) and II(t) indicate the waveforms of inductions I and II, respectively.

\begin{equation*} \mathrm {aVR}\left ({\mathrm {t} }\right)=-\left ({\frac {\mathrm {I}\left ({\mathrm {t} }\right)\mathrm {+II(t)}}{2} }\right) \tag{1}\end{equation*}

**FIGURE 3.**
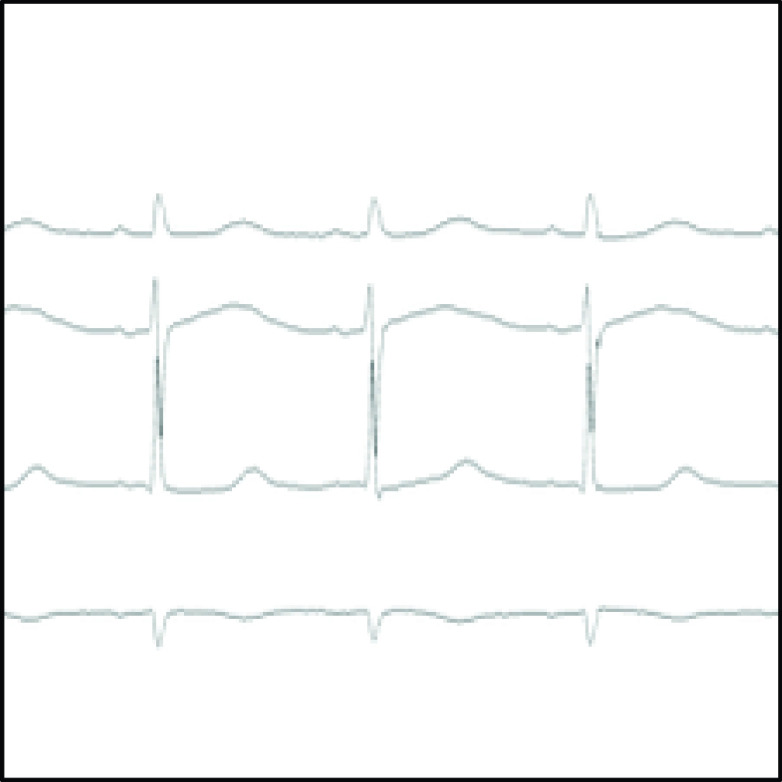
ECG image (PNG $224\times 224$ pixels).

After calculating the aVR(t) values for 5000 points, the potentials for the 5000 points stored in the CSV file were plotted on the image. Linear interpolation between the points was used to represent the ECG waveform with continuity to the plotted points. Cardiologists check the rhythm and then the shape on the ECG waveform when deciphering the ECG waveform. To get closer to the clinical ECG waveform, it is necessary to draw the ECG waveform with a time resolution that allows the rhythm and shape of the ECG waveform to be read, so the 5000 potentials were divided into three parts and converted into three images. To reduce the matrix size to $224\times224$ when the image was given to the CNN, the Lanczos function was used to reduce the image size to an 8-bit grayscale image. Subsequently, the inductions used in this study were converted to a single image by drawing four inductions in the order of II, V3, V5, and aVR from top to bottom, with 40 pixels between each induction. Figure 3 shows the converted images.

### D. CNN Architecture

In this study, ECG patterns divided into three images per case were given to the CNN to classify them as survivor and non-survivor. The CNN should be trained with a sufficient number of images to obtain good processing performance, and a small number of images may result in over-fitting.

Transfer learning is a method for creating CNN models with favorable performance using a small number of images. Several CNN models have been proposed that have been trained on a large amount of general image data, not including medical images, and have been confirmed to have excellent processing performance. Transfer learning reuses most of the structure and parameters of pre-trained CNN models and transfers them to other tasks.

In this study, seven CNNs, InceptionV3, ResNet50, VGG16/19, and DenseNet121/169/201, which were pre-trained with Image Net, a large natural image dataset, were introduced as the classification models. Consequently, transfer learning was performed by deleting the final convolutional layer beyond the pre-trained CNN model and combining the two layers to learn only all the coupled layers.

### E. Comprehensive Predictions for Patients

The CNN survival prediction results were obtained independently for each of the three images per patient, and the probability of death for each image was obtained as a continuous value. The final prediction result requires the integration of these three results. Therefore, the average of the three continuous values (probability of death) was used as the overall evaluation and prediction result.

### F. Visualization of CNN Decision Basis With GradCAM

When cardiologists interpret ECGs, they focus on the shape and temporal changes of the waveforms. In the analysis of ECGs by CNN, it is important to analyze which part of the waveform was analyzed while paying attention to the identification of the waveform. In this study, GradCAM was used to visualize the basis of the CNN decisions. First, electrocardiogram data were input into a trained CNN model to predict death and survival. The gradient of the final convolutional layer of the network was then used to obtain a heat map to visualize the areas on which the predictions were based. In this heatmap, the areas with large gradients are colored red, indicating that the CNN performed strong convolution in those regions, whereas the blue areas with small gradients indicate that the CNN performed weak convolution.

### G. Validation

#### 1) 10-Fold Cross-Validation

**FIGURE 4.**
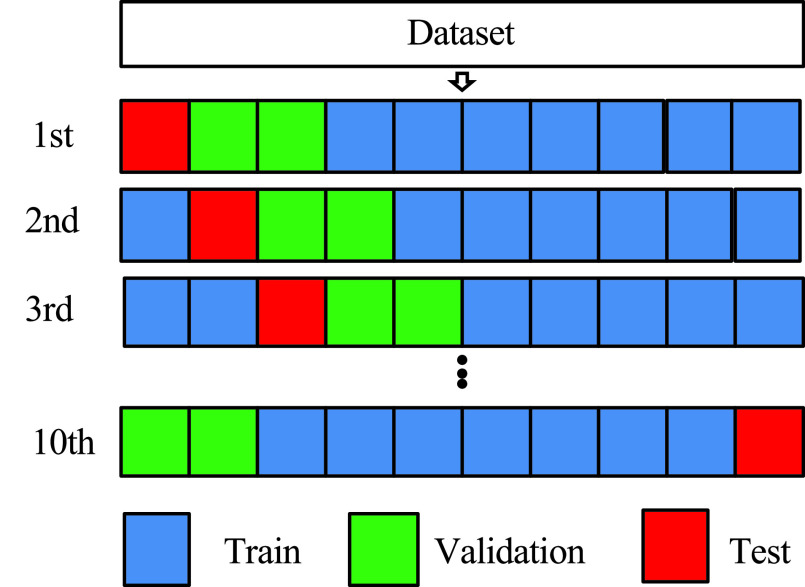
Ten-fold cross validation.

A ten-fold cross-validation was used to validate the prediction accuracy of the trained CNN models. Figure 4 shows a schematic of the 10-fold cross-validation. First, all the datasets were divided into 10 groups. One of the ten groups was used for the test. The remaining seven groups were used for training, and two for validation. The test data set was used to calculate the prediction accuracy. The parameters were then initialized to those previously trained by Image Net and repeated 10 times so that all the split groups became test data, and the average of the prediction accuracy of the 10 patterns was calculated as the prediction accuracy of this method. In this study, 406 cases per fold were used for CNN training, 116 for validation, and 60 for testing. The larger the number of splits in the cross-validation method, the less variation there is in the prediction results. In this study, the number of folds was set to 10 in consideration of the calculation cost for the number of samples.

#### 2) Evaluation Items

To evaluate the accuracy of the prediction, the number of true positives (TPs), true negatives (TNs), false positives (FPs), and false negatives (FNs) was calculated from the CNN classification results, and the sensitivity, specificity, and percentage of correct responses were calculated and used for evaluation. The calculation formulas are shown in (2) to (4): The area under the curve (AUC), which represents the area under the receiver operating characteristic (ROC) curve, was also calculated. ROC curves were obtained by plotting the false-positive rate on the horizontal axis (from 0 to 1) and the sensitivity on the vertical axis (from 0 to 1) against the continuous values obtained by the CNN and continuously varying the cutoff value (the reference value for the test).

\begin{align*} \mathrm {Sensitivity} &=\frac {\mathrm {TPs}}{\mathrm {TPs+FNs}}\times 100\left [{ \% }\right] \tag{2}\\ \mathrm {Specificity} &=\frac {\mathrm {TNs}}{\mathrm {FPs+TNs}}\times 100\left [{ \% }\right] \tag{3}\\ \mathrm {Accuracy} &=\frac {\mathrm {TPs+TNs}}{\mathrm {TPs+TNs+FPs+FNs}}\times 100\left [{ \% }\right] \tag{4}\end{align*}

#### 3) Learning Environments and Conditions

In this study, Keras and TensorFlow were used to create CNN models, and a PC with an NVIDIA Quadro RTX8000 GPU and an Intel Xeon E5-1650 CPU was used. The CNN was trained with a learning rate of $1\times 10^{-6}$, 50 training epochs, and batch size of 8.

## III. Results

**FIGURE 5.**
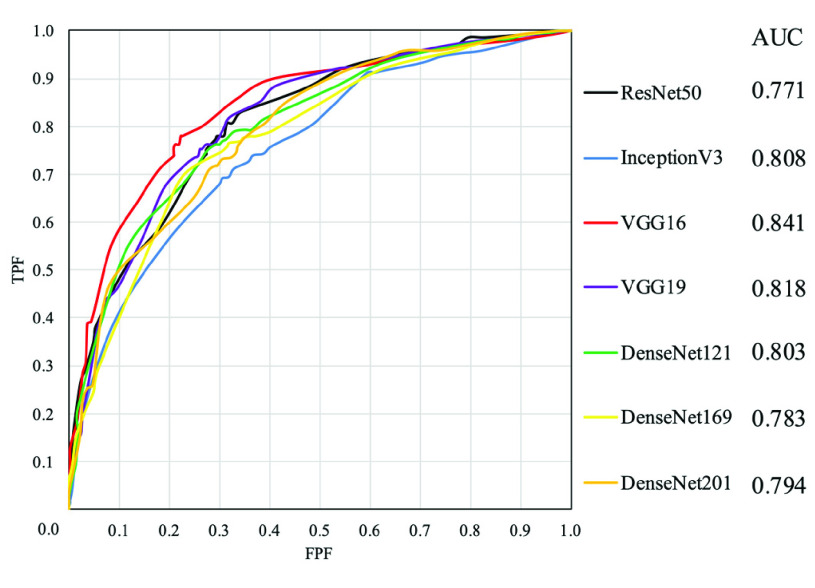
ROC curves with seven CNNs.

**FIGURE 6.**
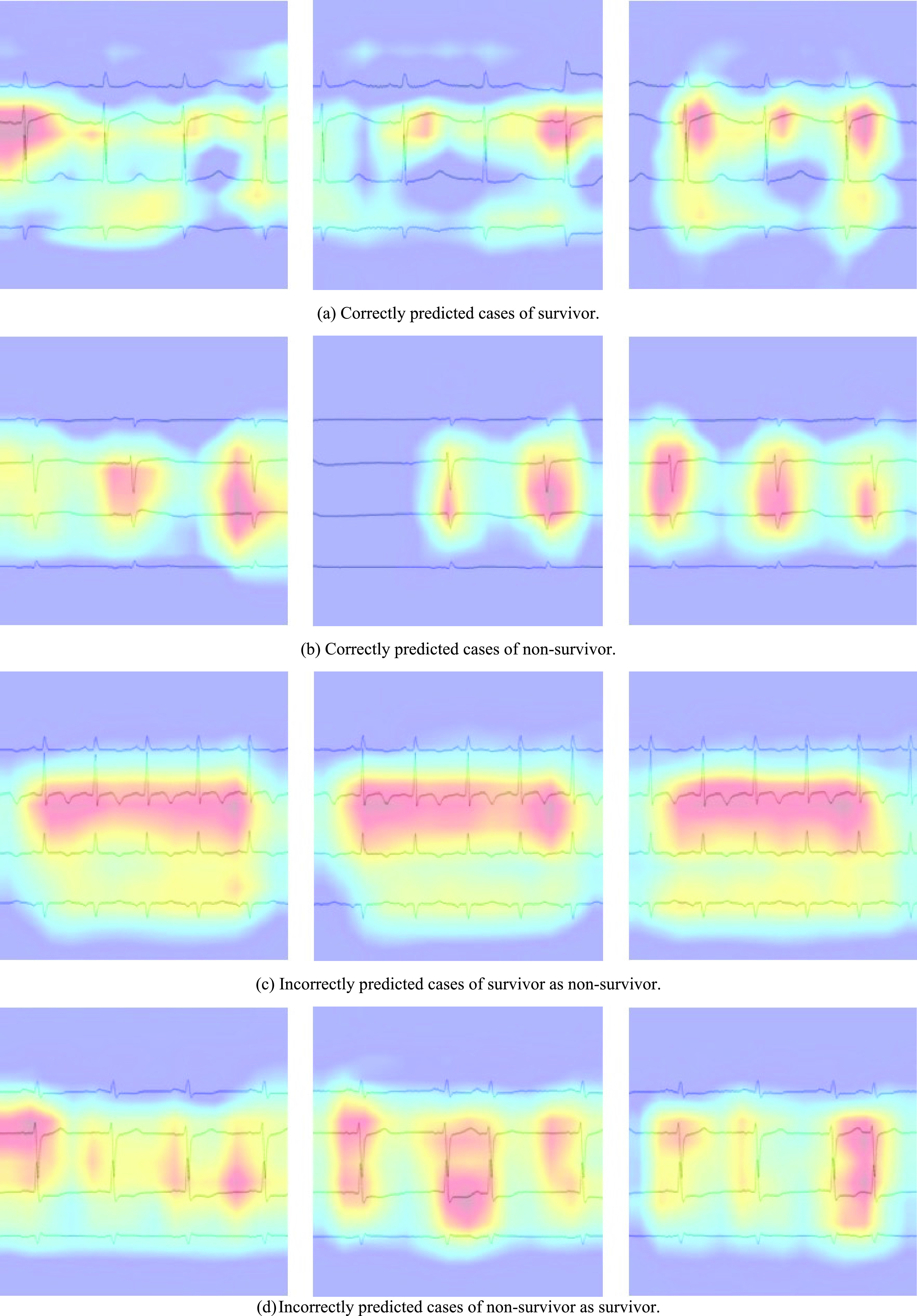
An example of a CNN heatmap obtained from GradCAM.

The prediction results for each CNN model are listed in Table 2. Table 3 lists the confusion matrices of the survival and death prediction results for the seven CNNs. Figure 5 shows the ROC curves for each CNN. Figure 6 shows the heat maps obtained by the Grad CAM with VGG16 overlaid on the ECG image.

**TABLE 2:** Prediction Results for Each CNN Model

	Accuracy	Sensitivity	Specificity	AUC
InceptionV3	69.1 %	71.1 %	67.0 %	0.771
ResNet50	74.1 %	77.3 %	70.8 %	0.808
VGG16	77.3 %	75.6 %	79.0 %	0.841
VGG19	74.6 %	75.3 %	73.9 %	0.818
DenseNet121	73.2 %	77.0 %	69.4 %	0.803
DenseNet169	72.3 %	76.6 %	68.0 %	0.783
DenseNet201	71.3 %	73.2 %	69.4 %	0.794

**TABLE 3:** Confusion Matrixes for Each CNN Model

(a) InceptionV3			
		Prediction	
		Non-survivor	Survivor
Actual	Non-survivor	219	72
	Survivor	76	215
(b) ResNet50			
		Prediction	
		Non-survivor	Survivor
Actual	Non-survivor	207	84
	Survivor	96	195
(c) VGG16			
		Prediction	
		Non-survivor	Survivor
Actual	Non-survivor	220	71
	Survivor	61	230
(d) VGG19			
		Prediction	
		Non-survivor	Survivor
Actual	Non-survivor	223	68
	Survivor	93	198
(e) DenseNet121			
		Prediction	
		Non-survivor	Survivor
Actual	Non-survivor	213	78
	Survivor	89	202
(f) DenseNet169			
		Prediction	
		Non-survivor	Survivor
Actual	Non-survivor	224	67
	Survivor	89	202
(g) DenseNet201			
		Prediction	
		Non-survivor	Survivor
Actual	Non-survivor	220	71
	Survivor	61	230

## IV. Discussion

In this study, we developed a method to predict short-term prognosis using CNN in 2D ECG images of patients in the CCU and analyzed the basis for CNN decisions. The CNN model was pretrained using Image Net, and the prediction accuracy was compared among seven different CNNs. When VGG16 was used, the accuracy, sensitivity, specificity, and AUC were 77.3%, 75.6%, 79.0%, and 0.841, respectively. VGG16 showed the highest prediction accuracy of all evaluation items. Because the network structure of VGG16 has fewer layers than the other CNN models used in this study. It is assumed that the highest accuracy was obtained because it could extract features that are important for prognosis prediction from ECG waveforms with multiple inductions. Compared to the results of Raghunath et al. [26], the task is completely different because it was one-year mortality. However, in the short-term prognosis of mortality, where patients’ symptoms are likely to change, accuracy was 77.3% and AUC was 0.841. These results are deemed to be agreeable.

In addition, to confirm the effectiveness of the proposed method, we compared the accuracy with one-dimensional CNN, which is used in conventional ECG waveform analysis. The same four ECG inductions (II, V3, V5, aVR) as in the proposed methods were input to independent 1DCNNs, and their features were combined and classified in a fully connected layer [35]. The 1DCNN consists of four convolutional layers and pooling layers. As a result of evaluation, the accuracy, sensitivity, specificity, and AUC were 69.0%, 61.7%, 76.2%, and 0.763, respectively. VGG16, a two-dimensional CNN, showed superior results in all evaluation items. Therefore, the two-dimensional analysis of the ECG waveforms was able to extract more prognostically effective features in CCU patients than the one-dimensional analysis.

GradCAM was used to obtain a heat map showing the basis for the decision of the CNN. Figure 6 (a) focuses on the S-T portion of V3 induction for patients with S-T changes in cases where patient survival was correctly predicted. (b) In cases in which death was correctly predicted, CNN attention was focused on the R-wave height in V3 and V5 induction in patients with left ventricular hypertrophy. Additionally, the case shown in (c), in which a surviving patient was incorrectly predicted to be a non-survivor, produced a continuous heat map in the V3 induction for the tachycardia case. In case (d), in which a surviving patient was incorrectly predicted to die, the CNN focused on the waveform of the extrasystoles in the left and middle images. From these results, it was confirmed that CNN tends to focus on the shape and regularity of waveforms and predict them.

This study has a limitation. In this study, 291 cases were randomly sampled from a survivor group of 9457 cases. Future work should be done on all survivor patients to evaluate the generalization performance of this approach.

The translational aspect of this study is that the system uses routine ECGs and can easily incorporate highly accurate AI models into clinical routines. Patients identified as high risk can be matched to GradCAM outputs for prompt medical therapy or aggressive intervention with an implantable device. This method is useful for these risk stratifications. This reduces the burden on the clinical staff as well as cardiologists and may lead to the detection of fatal ECG waveforms.

## V. Conclusion

In this study, we developed a 2D CNN-based short-term prognosis prediction and GradCAM-based explainable technique using electrocardiogram images of patients admitted to the CCU. The correct response rate, sensitivity, specificity, and area under the ROC curve for the CCU inpatients were 77.3 %, 75.6 %, 79.0 %, and 0.841, respectively. These results are good considering the difficulty of predictive processing using ECG waveforms in CCU patients. The visualization of the CNN by GradCAM showed that CNN tended to focus on the regularity of the ECG waveforms and their shape. These results suggest that our method identifies patients at high risk, who can then be given more intensive attention and treatment.
